# Relationship between Self-Perception of Aging and Quality of Life in the Different Stages of Reproductive Aging in Mexican Women

**DOI:** 10.3390/ijerph19116839

**Published:** 2022-06-02

**Authors:** Frida Sara Rivera-Ochoa, Ixel Venecia González-Herrera, Mariano Zacarías-Flores, Elsa Correa-Muñoz, Víctor Manuel Mendoza-Núñez, Martha A. Sánchez-Rodríguez

**Affiliations:** 1 Research Unit on Gerontology, FES Zaragoza, National Autonomous University of Mexico, Av. Guelatao No. 66, Col. Ejército de Oriente, Iztapalapa, Ciudad de Mexico CP 09230, Mexico; fridarivera94@gmail.com (F.S.R.-O.); ixelgonzalez@gmail.com (I.V.G.-H.); elcomm_unam@yahoo.com.mx (E.C.-M.); mendovic@unam.mx (V.M.M.-N.); 2 Division of Obstetrics and Gynecology, Hospital Gustavo Baz Prada, Institute of Health of the State of Mexico, Nezahualcóyotl, Estado de Mexico CP 57300, Mexico; mzacariasf@yahoo.com

**Keywords:** female aging, aging self-perception, quality of life, STRAW

## Abstract

Biological aging has an abrupt beginning in women, changing their body and perceptions, which are not accepted easily because the actual stereotypes are focused on youth and anti-aging. Our interest was to explore what the self-perception of aging (SPA) is in middle-aged women throughout the reproductive aging stages and their association with the quality of life. A cross-sectional study was conducted with 240 women (40–69 years) living in Mexico City, who were separated according to their reproductive aging stage. An electronic version of the Spanish version of the Self-rated Attitudes Towards Old Age (SATO) and the WHO Quality of Life-Bref (WHOQoL) was applied to these women and was sent by WhatsApp or email. Seventeen women of the total sample (7%) had a negative self-perception of aging. There is an association between SATO and WHOQoL (r = –0.273, *p* < 0.0001), but in the menopausal transition stage, the association is strong in the psychological subscale, and after menopause, early and late postmenopausal women show a better association in the social subscale. Negative SPA impacts the WHOQoL psychological dimension and not the total WHOQoL score. Our findings suggest an association between SPA and quality of life in different reproductive aging stages.

## 1. Introduction

The onset of female aging is associated with ovarian senescence, which causes the permanent cessation of menses due to the decreased production of estrogens and androgens, with the estrogen depletion being the most important event [1]. This process modifies woman’s metabolism causing physical, psychological, and social changes, with vasomotor, urogenital, sexual, central nervous and cardiovascular system alterations [1,2]. It is estimated that more than 50% of postmenopausal women suffer from moderate to severe symptoms which not only produce a biological effect, but also have psychological repercussions that affect the mood and quality of life [3,4]. As it is known, the menopausal transition starts before 50 years of age, and in Mexican women, 67% have vasomotor symptoms and 54% mood disorders such as irritability and nervousness [5]; thus, the presence of symptoms is dependent on the stage of reproductive aging in which women are. To understand the process, reproductive aging is separated into stages, and “*The Stages of Reproductive Aging Workshop”* + 10 (STRAW) is the classification that is more accepted. According to this classification, the menopausal transition stage is the period in which women have variable lengths of menstrual cycles and intervals of amenorrhea, before the menopause. Menopause is the moment after 12 months of amenorrhea, and the postmenopause period is separated into two stages, the early postmenopause until 6 years after the menopause and late postmenopause after 6 years of the menopause and for the remaining lifespan [6].

In addition, an increase in central obesity [7,8] and the initial signs of aging in body and skin changes are also present during the menopausal transition and postmenopausal periods, which are not accepted easily by women because the actual stereotypes are focused on youth and anti-aging [9]. As it is highlighted above, the fluctuations of sex hormones during menopausal stages cause mood changes, mainly depression and anxiety. These mood changes are favored by other stressful life factors such as physical and social changes, with Hispanic women being more likely to suffer from them [2,10], although more studies are necessary to clarify which of these factors are directly related [10].

Although the female body image is a psychological problem in all stages of life, in the postmenopausal period it has become a cause of malaise, distress, loss of identity, and low self-esteem, thus impacting their quality of life, as several qualitative studies have reported. Additionally, the stereotypes of age and the self-perception of aging (SPA) worsen the physical perception [9,11], which may be due to ageism.

Ageism involves prejudice, discrimination, and stereotype, and focuses predominantly on the negative aspects of aging [12]. It is acknowledged that the middle-age is the stage in life which concerns future old age. [13], because older age is typecast as a decline in physical and mental capacities with a high possibility of disability [14].

The negative attitudes to aging are associated with adverse events in health, highlighting their relationship with depression, hopelessness, anxiety, and slower recovery from disability [15,16,17,18]. Likewise, the negative information about aging has an influence on the physical perception of age. Thus, in an intervention study, the subjects who received negative information about aging felt threatened and perceived themselves as being physically older than their chronological age with more negative self-perception [19]. All those aspects affect the quality of life and health.

In this context, most of the studies on SPA have been conducted in older adults, and very little research has been done in middle-aged people. If we consider that in women biological aging is related to menopause and that unpleasant physical and psychological changes can occur at different stages of female aging, which can affect their quality of life, it is possible that the SPA and quality of life may be different depending on their stage; however, there are no studies about this topic. Thus, the aim of this study was to determine the association between SPA and quality of life in the different stages of reproductive aging in Mexican women.

## 2. Materials and Methods

### 2.1. Study Design and Population

A community-based cross-sectional study was conducted with 40–69-year-old women from Mexico City, Mexico. They were invited to participate in the project “Menopause and oxidative stress” directed by the Gerontology Research Unit at Universidad Nacional Autonoma de Mexico, Zaragoza Campus, from March to October 2021. The sample consisted of 240 women recruited by email and WhatsApp and separated according to their reproductive aging into three groups: (a) 93 menopausal transition women, (b) 80 early postmenopausal women, and (c) 67 late postmenopausal women. The eligibility criteria for menopausal transition women were that they still have menstrual periods; the early postmenopausal women were those that had at least 12 months of spontaneous amenorrhea and until 6 years after menopause; the late postmenopausal women were those that were more than 6 years after menopause [6]. All the women were free of cardiovascular, kidney, and cancer diseases, and had a minimum primary education and access to a smartphone, computer, or tablet.

An electronic version of the questionnaire using Google Forms was performed separated into four sections. The first section contained the notice of privacy and the informed consent. The second section consisted of general data, such as name, date of birth, highest level of education, years of school, marital status, date of last menses, years after last menstrual period (the value 0 was used by the menopausal transition women), and gynecological questions; this information was used to classify the women. In the third section, there was a transcript of the Spanish version of the Self-rated Attitudes Towards Old Age (SATO), a questionnaire developed by the University of the State of Mexico. Finally, the Spanish version of the WHO Quality of Life-Bref (WHOQoL) was in the last section. This form was sent by WhatsApp and email to the selected participants once.

All women were required to accept a digital informed consent; if they did not agree, they could not answer the questionnaires. This work is part of a clinical trial approved by the Ethics Committee of the National Autonomous University of Mexico (UNAM) Zaragoza Campus (register number FESZ/DEPI/CI/096/19).

### 2.2. Sample Size

The sample size was calculated based on the correlation point biserial two-tail model using G-Power software (Heinrich Heine University of Dusseldorf, Dusseldorf, Germany) on the assumption of the relationship between quality of life and the negative consequences of aging perception (Spearman r_s_ = −0.37), as it was previously reported [20]. We used a 5% of significance level and a power of 90%. The final sample size obtained was 67 women by a group, but to ensure accurate results, we included all the women that wanted to participate and met the eligibility criteria by each group as convenience samples.

### 2.3. Assessment of Self-Perception of Aging

The SATO has 21 items in a self-reported questionnaire format that explores three subscales: negative physical and behavioral stereotypes toward old age (negative stereotype), fear of aging itself (fear of aging), and fear of intellectual decline and abandonment (fear of decline) [Supplementary Material Table S1]. The instrument was originally validated and revised by gerontology experts [21]. The reliability of the electronic version was α = 0.900, and intraclass correlation coefficient = 0.708, *p* < 0.0001.

Each item can be graded from 1 (completely disagree) to 4 (completely agree); the total score is the sum of the scores obtained for all items and ranging from 21 to 84. A high score indicates negative self-perception. A cutoff value ≤ 44 was considered to indicate positive self-perception, and a score ≥ 59 classifies the women with a negative self-perception [22].

### 2.4. Assessment of Quality of Life+

The WHOQoL is a self-report questionnaire that includes 26 items in four subscales: physical health (physical), psychological health (psychological), social relationships (social), and environment functioning (environment); there are two global questions regarding quality of life and overall health. Each item can be assessed from 1 (very dissatisfied/not at all) to 5 (very satisfied/completely), the total ranges from 26 to 130, and higher scores show a good quality of life [23]. The reliability of the electronic version was α = 0.942, and intraclass correlation coefficient = 0.794, *p* < 0.0001.

The quality of life is classified as bad, average, and good according to the different cutoff values for each subscale and the total score [24].

### 2.5. Data Analysis

Quantitative data were expressed as the mean ± standard deviation, and these were compared with one-way ANOVA and Dunnett’s test post hoc using menopausal transition women as the control group. Frequencies and percentages were calculated for categorical data, and the differences were evaluated using the chi squared test and 95% confidence intervals. The frequencies of positive/negative SPA for each ordinal subscale of the quality of life were obtained.

Pearson’s correlation analysis was calculated to assess the association between WHOQoL with their subscales and SATO scores, obtaining the relationship for each of the three groups: menopausal transition, early postmenopausal, and late postmenopausal women. Multiple linear regression analysis was performed to examine the association between the WHOQoL total score as the dependent variable and SATO score and the control variables (covariates): age, years of school, and years after last menopausal period. These covariates were selected to analyze if they were correlated with WHOQoL and potentially could modify the primary association.

A two-tailed p-value lower than 0.05 was considered statistically significant. The data were processed using the standard statistical software package SPSS V. 26.0 (IBM SPSS Statistics, Armonk, NY, USA).

## 3. Results

### 3.1. Self-Perception of Aging and Quality of Life

We invited 250 women to participate in the study, and 240 (96%) of them responded. The groups only differ in age and the frequency of hysterectomy. Most of the women were married, and their highest educational level was university (Table 1).

Out of all the women, 152 (63%) considered their quality of life to be good and 17 (7%) had a negative self-perception of aging. Stratified by the reproductive aging stage, the score in the environment subscale is higher in late postmenopausal women compared with menopausal transition women (*p* < 0.05) (Table 2).

**Figure 1:**
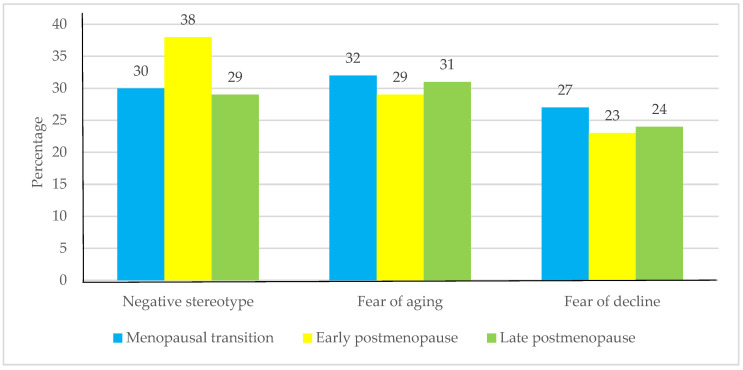
Prevalence of self-perception of aging subscales stratified by reproductive aging stages.

The early postmenopausal women’s group self-reported the highest percentage of a “negative stereotype perception” of aging (38%), which decreased in the late postmenopausal group (29%), although the difference was not statistically significant. Likewise, the percentage of self-reported “fear of decline perception” decreased marginally from the menopausal transition group (27%) to the early postmenopausal group (23%) and the late postmenopausal group (24%) (Figure 1).

### 3.2. Association between Self-Perception of Aging and Quality of Life

**Figure 2:**
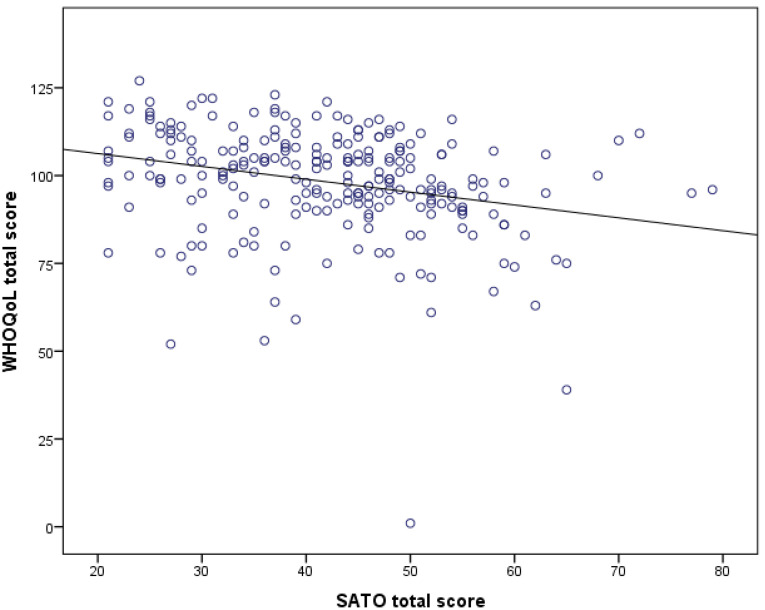
Linear association between WHO Quality of Life (WHOQoL) total score and Self-perception Attitudes Towards Old Age (SATO) total score. Pearson’s correlation, r = −0.273, *p* < 0.0001.

There is a linear weak, but significant, negative association between WHOQoL and SATO scores (r = −0.273, *p* < 0.0001) (Figure 2).

Stratifying by the reproductive aging stage, the correlation between WHOQoL and SATO is higher in the late postmenopausal women than the other groups (r = −0.307, *p* < 0.01). Regarding the subscales, all correlations are statistically significant in the menopausal transition women, revealing that the psychological subscale has the highest association (r= −0.282, *p* < 0.01). In the early postmenopausal group, the physical subscale has no association, and the social subscale shows a better relation (r= −0.263, *p* < 0. 01). Additionally, in the late postmenopausal group, this subscale has the highest association (r= −0.385, *p* < 0.01) (Table 3).

In the multivariate analysis, the years of school is a covariate that is statistically significant in the association between quality of life and self-perception of aging, the other covariates are not related. Thus, by each year of school, the WHOQoL total score increases by 0.76 points, and by each point of the SATO score, the WHOQoL reduces by 0.31 points (Table 4).

**Figure 3:**
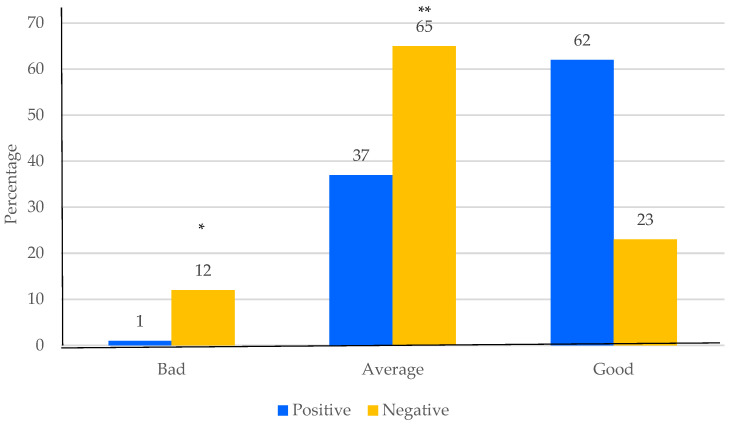
Frequency of self-perception of aging with different levels of quality of life in the psychological subscale. Chi squared test * bad vs. good quality of life, *p* < 0.001; ** average vs. good quality of life, *p* < 0.05.

In the psychological subscale, negative self-perception is more frequent than positive perception in the women with a bad quality of life, as well as in the women with an average quality of life. In contrast, the women with a good quality of life have a positive self-perception of aging (Figure 3).

## 4. Discussion

Aging is a natural process of humans and has its differences between women and men. The physiological changes in women are more abrupt and they happen around menopause, with psychological and social repercussions [2]. Most of the time, these changes produce unpleasant sensations as they change from a reproductive to a non-reproductive stage, which is also the beginning of aging, and women usually have a bad perception about this. Moreover, current stereotypes are anti-aging; therefore, there is a rejection of aging and an implicit ageism, with the idea of remaining young in appearance and behavior. It has become a common thought of women [9,25].

In this study, most women in the three stages of reproductive aging (≥59%) acknowledged having a good quality of life and only 2% in each of these groups self-reported a bad quality of life. Likewise, the majority (≥55%) of the women in the three groups have a positive perception of aging and only 4 to 10% have a negative perception of aging. In this sense, it has been described in different qualitative studies that middle-aged women have contrasting opinions about their bio-physiological changes, some consider it to be a time of decline, while others think it is a stage of a new beginning, accepting their new social role [11,26]. Then, if women have a positive view of menopausal changes, a positive SPA will also be favored. A recent report found a direct effect of the positive perception of aging on the quality of life; although the study population was above 55 years of age (mean age 66.9 years), these findings are comparable with our study [20].

Regarding the SPA, the SATO allows it to be analyzed in three dimensions; of these, the results showed that only the negative stereotype is a concern in early postmenopausal women, similar to the results of a study which indicates that people between 50 and 59 years, regardless of gender, feel they experience unfair treatment more than older people, although these experiences are not considered age discrimination [27]. This is possible because, as with older people, early postmenopausal women incorporate an age-stereotype in such a way that they perceive themselves as an aged person, thus accepting these traits [28,29].

The scales of the instruments used to measure the variables of interest are opposing; a higher WHOQoL score indicates a good quality of life, and a lower SATO score shows the perception of aging as positive. Like the SATO, the WHOQoL evaluates four dimensions which also have a negative correlation with the perception of aging, but the better association is different for each subscale in each reproductive aging stage. Thus, in the menopausal transition stage, the association is strong in the psychological subscale, showing that the feelings and self-acceptance are focused on the lost youth, having a negative perception of aging with feelings of depression and denial [26]. This is possible because the women in this stage have several physical and psychological symptoms that cause stress [30], modifying their self-perception and self-esteem [11].

After menopause, both groups, early and late postmenopausal women, show a better association in the social subscale, that is, negative SPA related to the bad social quality of life, mainly in the late postmenopausal group. Although we were not able to find previous reports about this relationship, an investigation with Mexican postmenopausal women reported 67% of the sample with a low quality of life in the social dimension and high oxidative stress; hence, a poor quality of life in the social environment has a direct impact on the body [31]. It is known that at this time of life there are changes in family life and social relationships; the role of a woman in her family becomes less important, and the occupational status may change several times, leaving the workforce to return to the activities at home, which causes discomfort in some women. Various studies point out that the postmenopausal women who have unfavorable changes in their life and poor psychosocial and occupational functioning will have a bad quality of life [32,33] and consequently a negative SPA. Nevertheless, de Salis et al., cited that most women in the world consider menopause as a passing state, an opportunity for a new life beyond the reproductive stage [11].

On the other hand, the quality of life and SPA relationship is affected by the years of formal education, but not by age and years after the last menstrual period. The experience of menopausal symptoms is related to a low educational level and poor quality of life, perhaps related to the expectations about menopause that women with low education have, given that women with higher levels of education have less severe symptoms on many occasions [34]. Higher levels of education increase the chances of a better quality of life and health when women are older [35]. In contrast to this, a cohort study with subjects aged 65 years and over found that there is no change in quality of life in relation to educational level, probably due to the acceptance of aging that translates into satisfaction with life, regardless of gender [36].

With respect to age, the information is contrasting; it is reported that middle-aged women have a poor quality of life, especially while they are in the early postmenopausal period, probably due to the severity of menopausal symptoms [37]. Another study clarifies that in older woman the symptoms decrease and therefore the quality of life improves [38]; however, in our work, neither of the two effects can be demonstrated, even adjusting for years after the last menstrual period. The inconsistency between our results and the reports indicated may be due to the instruments used. In both studies, the MENQOL questionnaire was used, and it focuses on the severity of menopausal symptoms, and how it affects women’s lives [37,38]. The WHOQoL used in our study is a more comprehensive instrument to assess the quality of life, not just menopausal symptoms; although the aim of both questionnaires is the quality of life, the focus of each questionnaire is different.

Likewise, women with a bad or average quality of life in the psychological dimension of WHOQoL have a negative SPA, which shows that negative perceptions impact this dimension, and it does not change the evaluation of the total score. A study analyzing psychological and psychosocial determinants of quality of life in aged individuals found that the psychological distress is related to quality of life and the negative perception of aging [20]. Moreover, several studies have shown that negative SPA and negative stereotypes are stronger related with deleterious effects to health and hopelessness, such as depression, anxiety, cardiovascular events, and obesity [15,16,17,39]. A systematic review of the literature which explores the SPA of older adults and their health found that more negative attitudes are associated with a lower quality of life and worse health status, especially mental status; the older adults with a high quality of life had more positive aging attitudes [40].

It is important to understand that a woman’s reproductive aging is another stage in her life, a new cycle with new opportunities and challenges to prepare a good quality of life in older age [41], leaving negative stereotypes aside, as has been noted in studies with older adults [42]. Most of the women in this study perceive their quality of life as good possibly because they are accepting this stage, hence the low prevalence of negative self-perception of aging. These results are consistent with those reported by Sun et al., (2022) who found an association between self-perceived stigma and quality of life in urban Chinese older adults [43]. Despite the similarity in the results, it is important to note that our study is the first to address this relationship in middle-aged women, exploring the process of female aging. The aim of our research was to identify factors that have an impact on quality of life. With these results, we can see that the negative perception of aging affects the psychological dimension and that the quality of life in general apparently does not change during female reproductive aging, although further studies are needed to corroborate our findings.

On the other hand, recently the concept of “profiguration” has been incorporated in the field of gerontology, which considers the promotion and strengthening of intergenerational interdependence, education, wellbeing, social participation, and active aging as a key element for quality of life and overcoming ageism. Profiguration is characterized by a participatory, cooperative, and comprehensive education to promote social change through collaborative and inclusive decisions of interactions between several generations and gender equality. Furthermore, profigurative cultures and societies create interdependence among people at different stages of life, thus avoiding loneliness and ageism. This approach can be integrated into the study of the aging of female reproduction to improve the quality of life and reduce ageism among women. In this regard, the commitment of all members of the community, young and old, men and women, is essential for social development. Therefore, “profiguration” can mitigate and/or overcome the existing generation gap, improving coexistence and relationships between individuals of different ages and genders [44,45].

The sample size and a convenience sample, the use of the electronic version of the tests, and the cross-sectional design are limitations of this study; however, the homogeneity of the women selected, the compliance with the calculated sample size, and the analysis across the female reproductive aging are strengths of the study. Related to the sample selection, all the participants were volunteers, but there are no differences in the characteristics of the groups, which guarantees their comparability; nevertheless, studies with a larger sample size are necessary.

Regarding the use of the electronic version of the SATO and WHOQoL, the current pandemic situation worldwide prevents the application of face-to-face questionnaires, which is why this form of data collection was chosen. Thus, the accordance and reproducibility of the electronic questionnaires were obtained, and it was demonstrated that they are adequate and reliable, even with the possibility of bias that may exist in the responses due to the lack of supervision in the application. An earlier study demonstrated the reliability of the web version of WHOQoL-Bref for older adults [46], thus supporting evidence of its use. If this web version was applied to people with more digital limitations due to their age, the responses of middle-aged women participating in our study can be regarded as reliable. In addition, as access to digital devices was an inclusion criterion, then the participants must have the ability to handle these devices. In this context, we did not have reports of any problems in answering the questionnaires.

Finally, the cross-sectional design does not allow us to demonstrate a causal relationship, and the results cannot be generalized, but the association between the SPA and quality of life in the different reproductive aging stages was proven, allowing a causal relationship to be inferred. Nevertheless, it could be convenient to carry out longitudinal studies and in different cultural contexts to confirm our findings and seek possible differences.

Additionally, it is important to point out that in the “World Report on Aging and Health” (WHO, 2015) [47], female reproductive aging and its implications were not considered, which should be incorporated as a specific field of study of female healthy aging.

## 5. Conclusions

Our findings suggest that the negative self-perception of reproductive aging (self-stereotypes) has a negative influence on the quality of life of women in this stage of life, especially in the early postmenopausal period. Likewise, a low level of education is a factor associated with the negative self-perception of reproductive aging and quality of life. For this reason, in accordance with the provisions of the Global Report on Ageism (WHO, 2021) [48], it would be convenient to carry out more longitudinal studies on the effect of permanent campaigns to counteract ageism in different populations and contexts, including reproductive aging.

## Figures and Tables

**Table 1:** Description of study groups.

Variable	Menopausal Transition	Early Postmenopause	Late Postmenopause
	(*n* = 93)	(*n* = 80)	(*n* = 67)
Age (years)	46 ± 4	53 ± 4 *	59 ± 5 *
Marital status			
Single	13 (14%)	12 (15%)	14 (21%)
Married	58 (62%)	39 (49%)	38 (57%)
Separated/divorced	17 (18%)	17 (21%)	8 (12%)
Widow	5 (5%)	12 (15%)	7 (10%)
Educational level			
Primary school	0	2 (3%)	2 (3%)
Secondary school	11 (12%)	5 (6%)	6 (9%)
High school	31 (33%)	32 (40%)	19 (28%)
University	51 (55%)	41 (51%)	40 (60%)
Hysterectomy	5 (5%) ^†^	18 (23%)	17 (25%)
Oophorectomy	0	7 (9%)	5 (8%)

* One-way ANOVA with Dunnett’s test post hoc using menopausal transition group as control group, *p* < 0.0001; ^†^ chi squared test, *p* < 0.01.

**Table 2:** Self-perception of aging and the quality of life by study group.

Parameter	Menopausal Transition	Early Postmenopause	Late Postmenopause
	(*n* = 93)	(*n* = 80)	(*n* = 67)
Self-perception of aging			
Total score	42.1 ± 10.6	42.1 ± 11.9	41.6 ± 12.6
Positive perception (≤44)	51 (55%)	44 (55%)	39 (58%)
Negative perception (≥59)	4 (4%)	8 (10%)	5 (8%)
Quality of life score			
Total	97.5 ± 14.3	97.7 ± 14.8	99.9 ± 17.9
Physical subscale	26.5 ± 4.6	26.7 ± 4.7	26.5 ± 3.8
Psychological subscale	23.1 ± 3.7	22.8 ± 3.8	24.0 ± 6.8
Environment subscale	28.5 ± 5.2	29.5 ± 5.3	30.9 ± 6.4 *
Social subscale	11.7 ± 2.4	11.3 ± 2.4	12.0 ± 2.5
Quality of life classification			
Good (>95)	58 (62%)	47 (59%)	47 (70%)
Average (61–95)	33 (36%)	31 (39%)	19 (28%)
Bad (<65)	2 (2%)	2 (2%)	1 (2%)

* One-way ANOVA with Dunnett’s test post hoc, menopausal transition vs. late postmenopause, *p* < 0.05.

**Table 3:** Association between self-perception of aging and quality of life total score and their subscales, stratified by reproductive aging stage.

WHO Quality of Life	Menopausal Transition	Early Postmenopause	Late Postmenopause
Total score	−0.273 *	−0.237 ^†^	−0.307 *
Physical subscale	−0.212 ^†^	−0.121	−0.246 ^†^
Psychological subscale	−0.282 *	−0.232 ^†^	−0.059
Environment subscale	−0.226 ^†^	−0.241 ^†^	−0.293 *
Social subscale	−0.211 ^†^	−0.263 *	−0.385 *

Pearson’s correlation * *p* < 0.01, ^†^ *p* < 0.05.

**Table 4:** Multivariate association between quality of life and self-perception of aging with other covariates.

Variable	Beta	Standard Error	*p*-Value
SATO score	−0.31	0.05	<0.0001
Years of school	0.76	0.21	<0.0001
Age (years)	0.21	0.22	0.338
Years after last menstrual period	−0.16	0.31	0.590

Multiple linear regression, R = 0.353, *p* < 0.0001. SATO: Self-perception Attitudes Towards Old Age.

## Data Availability

The data used to support the findings of this study are available from the corresponding author upon request (masanrod@comunidad.unam.mx). All the participants have a key number; therefore, the risk of identification is very low.

## Supplementary Materials

The following supporting information can be downloaded at: https://www.mdpi.com/article/10.3390/ijerph19116839/s1, Supplementary Material Table S1: Self-rated Attitudes Towards Old Age Questionnaire.

## References

[1] Broekmans F.J., Soules M.R., Fauser B.C. (2009). Ovarian aging: Mechanisms and clinical consequences. Endocr. Rev..

[2] El Khoudary S.R., Greendale G., Crawford S.L., Avis N.E., Brooks M.M., Thurston R.C., Karvonen-Gutierrez C., Waetjen L.E., Matthews K. (2019). The menopause transition and women’s health at midlife: A progress report from the Study of Women’s Health Across the Nation (SWAN). Menopause.

[3] Masjoudi M., Amjadi M.A., Leyli E.K.N. (2017). Severity and frequency of menopausal symptoms in middle aged women, Rasht, Iran. J. Clin. Diagn. Res..

[4] Zhao D., Liu C., Feng X., Hou F., Xu X., Li P. (2019). Menopausal symptoms in different substages of perimenopause and their relationships with social support and resilience. Menopause.

[5] Hernández-Valencia M., Córdova-Pérez N., Basurto L., Saucedo R., Vargas C., Vargas A., Ruiz M., Manuel L., Zárate A. (2010). Frecuencia de los síntomas del síndrome climatérico. Ginecol. Obstetr. Mex..

[6] Harlow S.D., Gass M., Hall J.E., Lobo R., Maki P., Rebar R.W., Sherman S., Sluss P.M., de Villiers T.J., STRAW + 10 Collaborative Group (2012). Executive summary of the Stages of Reproductive Aging Workshop + 10: Addressing the unfinished agenda of staging reproductive aging. Menopause.

[7] Bitner D., Wild R. (2014). Clinical intervention to reduce central obesity and menopausal symptoms in women aged 35 to 55 years. Menopause.

[8] Rodríguez-San Nicolás A., Sánchez-Rodríguez M.A., Zacarías-Flores M., Correa-Muñoz E., Mendoza-Núñez V.M. (2020). Relación entre la obesidad central y el estrés oxidativo en mujeres premenopáusicas versus posmenopáusicas. Nutr. Hosp..

[9] Cameron E., Ward P., Mandville-Anstey S.A., Coombs A. (2019). The female aging body: A systematic review of female perspectives on aging, health, and body image. J. Women Aging.

[10] Cortés Y.I., Marginean V., Berry D. (2021). Physiologic and psychosocial changes of the menopause transition in US Latinas: A narrative review. Climacteric.

[11] de Salis I., Owen-Smith A., Donovan J.L., Lawlor D.A. (2018). Experiencing menopause in the UK: The interrelated narratives of normality, distress, and transformation. J. Women Aging..

[12] Officer A., de la Fuente-Núñez V. (2018). A global campaign to combat ageism. Bull. World Health Organ.

[13] Dolberg P., Ayalon L. (2018). Subjective meanings and identification with middle age. Int. J. Aging Human Dev..

[14] Palmore E.B. (2004). The Future of Ageism.

[15] Freeman A.T., Santini Z.I., Tyrovolas S., Rummel-Kluge C., Haro J.M., Koyanagi A. (2016). Negative perceptions of ageing predict the onset and persistence of depression and anxiety: Findings from a prospective analysis of the Irish Longitudinal Study on Ageing (TILDA). J. Affect. Disord..

[16] Gum A.M., Ayalon L. (2018). Self-perceptions of aging mediate the longitudinal relationship of hopelessness and depressive symptoms. Int. J. Geriatr. Psychiatry.

[17] Xiao L., Yang H., Du W., Lei H., Wang Z., Shao J. (2019). Subjective age and depressive symptoms among Chinese older adults: A moderated mediation model of perceived control and self-perceptions of aging. Psychiatry Res..

[18] Levy B.R., Slade M.D., Murphy T.E., Gill T.M. (2012). Association between positive age stereotypes and recovery from disability in older persons. JAMA.

[19] Marquet M., Boutaayamou M., Schwartz C., Locquet M., Bruyère O., Croisier J.L., Adam S. (2018). Does negative information about aging influence older adults’ physical performance and subjective age?. Arch. Gerontol. Geriatr..

[20] Ingrand I., Paccalin M., Liuu E., Gil R., Ingrand P. (2018). Positive perception of aging is a key predictor of quality-of-life in aging people. PLoS ONE.

[21] Hernández-Pozo M.R., Torres N.M., Coronado A.O., Herrera G.A., Castillo N.P., Sánchez V.A., González-Celis R.A.L. (2009). Actitudes negativas hacia la vejez en poblaci*ó*n mexicana: Aspectos psicom*é*tricos de una escala. Evaluación en Psicogerontología.

[22] Mendoza-Núñez V.M., Sarmiento-Salmorán E., Marín-Cortés R., Martínez-Maldonado M.L., Ruiz-Ramos M. (2018). Influence of the self-perception of old age on the effect of a Healthy Aging Program. J. Clin. Med..

[23] WHOQOL Group (1998). Development of the World Health Organization WHOQOLBREF quality of life assessment. Psychol. Med..

[24] Phungrassami T., Katikarn R., Watanaarepornchai S., Sangtawan D. (2004). Quality of life assessment in radiotherapy patients by WHOQOL-BREF-THAI: A feasibility study. J. Med. Assoc. Thai..

[25] Gewirtz-Meydan A., Ayalon L. (2018). Forever young: Visual representations of gender and age in online dating sites for older adults. J. Women Aging.

[26] Reyhani M., Kazemi A., Keshvari M. (2018). Rise and fall: Two sides of a coin of middle aged women’s perceptions of reproductive: A qualitative study. Arch. Womens Ment. Health.

[27] Giasson H.L., Queen T.L., Larkina M., Smith J. (2017). Age group differences in perceived age discrimination: Associations with self-perceptions of aging. Gerontologist.

[28] Levy B.R. (2003). Mind matters: Cognitive and physical effects of aging self-stereotypes. J. Gerontol. B Psychol. Sci. Soc. Sci..

[29] Levy B. (2009). Stereotype embodiment: A psychosocial approach to aging. Curr. Dir. Psychol. Sci..

[30] Binfa L., Castelo-Branco C., Blümel J.E., Cancelo M.J., Bonilla H., Muñoz I., Vergara V., Izaguirre H., Sarrá S., Ríos R.V. (2004). Influence of psycho-social factors on climacteric symptoms. Maturitas.

[31] Sánchez-Rodríguez M.A., Castrejón-Delgado L., Zacarías-Flores M., Arronte-Rosales A., Mendoza-Núñez V.M. (2017). Quality of life among post-menopausal women due to oxidative stress boosted by dysthymia and anxiety. BMC Women’s Health.

[32] Barnaś E., Penar-Zadarko B., Pikuła A. (2006). The assessment of women’s life quality during climacterium. Polish J. Environ. Stud..

[33] Robak-Chołubek D., Wdowiak A., Makara-Studzińska M., Korczyńska E. (2014). Perception and degree of acceptance of menopause-related changes in various spheres of life by postmenopausal women. Ann. Agric. Environ. Med..

[34] Karacam Z., Seker S.E. (2007). Factors associated with menopausal symptoms and their relationship with the quality of life among Turkish women. Maturitas.

[35] Krawczyk-Suszek M., Kleinrok A. (2022). Health-Related Quality of Life (HRQoL) of people over 65 years of age. Int. J. Environ. Res. Public Health.

[36] Abolhassani N., Santos-Eggimann B., Büla C., Goy R., Guessous I., Henchoz Y. (2019). Quality of life profile in three cohorts of community-dwelling Swiss older people. BMC Geriatrics.

[37] Ibrahim Z.M., Ghoneim H.M., Madny E., Kishk E.A., Lotfy M., Bahaa A., Taha T., Aboelroose A.A., Atwa K.A., Abbas A.M. (2020). The effect of menopausal symptoms on the quality of life among postmenopausal Egyptian women. Climacteric.

[38] Fallahzadeh H. (2010). Quality of life after the menopause in Iran: A population study. Qual. Life Res..

[39] Levy B.R., Slade M.D. (2019). Positive views of aging reduce risk of developing later-life obesity. Prev. Med. Rep..

[40] Warmoth K., Tarrant M., Abraham C., Lang I.A. (2016). Older adults’ perceptions of ageing and their health and functioning: A systematic review of observational studies. Psychol. Health Med..

[41] Moshki M., Mohammadzadeh F., Dehnoalian A. (2018). The effectiveness of a group-based educational program on the self-efficacy and self-acceptance of menopausal women: A randomized controlled trial. J. Women Aging..

[42] Kalfoss M.H. (2017). Attitudes to ageing among older Norwegian adults living in the community. Br. J. Community Nurs..

[43] Sun T., Zhang S.E., Yan M.Y., Lian T.H., Yu Y.Q., Yin H.Y., Zhao C.X., Wang Y.P., Chang X., Ji K.Y. (2022). Association between self-perceived stigma and quality of life among urban Chinese older adults: The moderating role of attitude toward own aging and traditionality. Front. Public Health.

[44] Molina-Luque F. (2020). The art of living as a community: Profiguration, sustainability, and social development in Rapa Nui. Sustainability.

[45] Molina-Luque F., Stončikaitė I., Torres-González T., Sanvicen-Torné P. (2022). Profiguration, active ageing, and creativity: Keys for quality of life and overcoming ageism. Int. J. Environ. Res. Public Health.

[46] Casamali F.F.C., Schuch F.B., Scortegagna S.A., Legnania E., De Marchi A.C.B. (2019). Accordance and reproducibility of the electronic version of the WHOQOLBREF and WHOQOL-OLD questionnaires. Exp. Gerontol..

[47] World Health Organization (2015). World Report on Ageing and Health.

[48] World Health Organization (2021). Promising strategies. Global Report on Ageism.

