# Supplementary material for: Relationship between Self-Perception of Aging and Quality of Life in the Different Stages of Reproductive Aging in Mexican Women
Source: Int J Environ Res Public Health. 2022 Jun 2;19(11):6839. doi: 10.3390/ijerph19116839 (PMC9180910; doi:10.3390/ijerph19116839)
Supplement: Supplementary file 1 [file ijerph-19-06839-s001.zip › ijerph-1668994-supplementary.pdf]

Supplementary material Table S1

Self-rated Attitudes Towards Old Age Questionnaire

Instructions: In this questionnaire you are asked how feel about ageing, considering negative physical and behavioral stereotypes, fear of aging itself, and fear of intellectual decline and abandonment. Select the level of agreement that you consider for each of the following statements.

		Completely disagree	Disagree	Agree	Completely agree
1	It makes me sad to be elderly.				
2	It is unpleasant to have wrinkles.				
3	Old age scares me.				
4	I do not like having graying hair.				
5	The elderly has bad breath.				
6	It is awful losing mental abilities with old age.				
7	Older people become fools and repeat themselves.				
8	With aging comes sadness and loneliness.				
9	Women with graying hair are unattractive.				
10	Older people are abandoned.				
11	Men with age-related baldness are unattractive.				
12	Elderly women should take steps to look younger.				
13	Being old is depressing.				
14	Older people are smelly.				
15	It is easy to fool old people.				
16	With aging, independence is lost.				
17	The elderly causes many problems.				
18	The elderly do not have skills such as driving cars.				
19	The elderly are greedy.				
20	Nursing homes are depressing.				
21	I fear being useless when I am old.				

Hernández-Pozo, M.R.; Torres, N.M.; Coronado, A.O.; Herrera, G.A.; Castillo, N.P.; Sánchez, V.A. Actitudes negativas hacia la vejez en población mexicana: aspectos psicométricos de una escala. In: *Evaluación en Psicogerontología*; González-Celis, R.A.L. Ed.; Manual Moderno: Mexico, 2009; pp. 1-16.
